# Draft Genome Sequences of Two *Marinitoga camini* Isolates Producing Bacterioviruses

**DOI:** 10.1128/genomeA.01261-16

**Published:** 2016-11-10

**Authors:** Coraline Mercier, Julien Lossouarn, Thomas Haverkamp, Nadège Bienvenu, Anne Godfroy, Valérie Cueff-Gauchard, Claire Geslin, Camilla Nesbo

**Affiliations:** a UBO, Laboratoire de Microbiologie des Environnements Extrêmes, UMR 6197, IUEM, Rue Dumont d’ Urville, Plouzané, France; b CNRS, Laboratoire de Microbiologie des Environnements Extrêmes, UMR 6197, IUEM, Rue Dumont d’Urville, Plouzané, France; c Ifremer, Laboratoire de Microbiologie des Environnements Extrêmes, UMR 6197, CS10070, Plouzané, France; d Centre for Ecological and Evolutionary Synthesis, University of Oslo, Oslo, Norway; e Department of Biological Sciences, University of Alberta, Edmonton, Alberta, Canada

## Abstract

Here, we present the draft genome sequences of two thermophilic *Marinitoga* strain members of the *Thermotogales* order, *Marinitoga camini* DV1155 and *Marinitoga camini* DV1197. These strains were isolated from deep-sea hydrothermal vents of the Mid-Atlantic Ridge.

## GENOME ANNOUNCEMENT

Bacteria from the *Marinitoga* genus are thermophilic, anaerobic, and organotrophic microorganisms isolated from various hot environments such as deep-sea hydrothermal vents or costal thermal springs. Among the five *Marinitoga* species described (1–5), only the *M. piezophila* KA3 genome has been sequenced (6). Here, we present the draft genome sequences of two novel *Marinitoga camini* isolates, DV1155 and DV1197, both containing proviral sequences.

The two strains were sampled during the DIVA 2 cruise in 1994 (7). *M. camini* DV1155 was isolated from a black smoker chimney at the Menez Gwen site. This hydrothermal field is located on the Mid-Atlantic Ridge at a depth of 840 to 870 m (8). *M. camini* DV1197 was isolated from a colonization module deployed at the Lucky strike hydrothermal vent field. This site is located at 1700 m of depth in the Mid-Atlantic Ridge (8). Strains DV1197 and DV1155 grow optimally at 60°C and 65°C, respectively, at atmospheric pressure in a modified Ravot medium (9) with elemental sulfur instead of cysteine.

Genomic DNA was extracted following the protocol of Geslin et al. (10). The purity and quantity of the DNA were measured using Nanodrop and Qubit instruments (Thermo, Fisher Scientific). Shotgun libraries were constructed using the Nextera XT kit and sequenced as one of ten pooled, barcoded libraries on a MiSeq (all from Illumina) using 500 cycles generating 2 × 250 bp paired-end reads. The genomes assembled *de novo* by CLC Genomics Workbench 7.0.4, using trimming default settings, automatic word size, a bubble size corresponding to the average length of the input reads, a minimum contig length of 1000 bp, and reads mapped back to the contigs.

For *M. camini* DV1155 this resulted in 56 contigs totaling 2,435,399 bp, with an *N*_50_ of 90,885 bp, longest contig size of 254.99 bp, and G+C content of 27.3%. No extrachromosomal DNA was observed but a proviral sequence of 50,700 pb was found in the genome using Prophinder (11). For *M. camini* DV1197, we obtained 51 contigs totaling 2,274,557 bp with an *N*_50_ of 72,669, longest contig size of 188,990 bp, and a G+C content of 27.4%. No extrachromosomal DNA was observed but a proviral sequence of 53,437 bp was detected using the same methods than for *M. camini* DV1155.

Both draft genomes were annotated in the NCBI Prokaryotic Genome Automatic Annotation Pipeline (PGAAP [12]), which identified 2,315 genes and 2,256 coding sequences (CDS) for *M. camini* DV1155 and 2,221 genes and 2,157 CDS for *M. camini* DV1197.

### Accession number(s).

Both whole-genome shotgun projects have been deposited at DDBJ/EMBL/GenBank under the accession no. AZAX00000000 and AZAY00000000 for *M. camini* DV1155 and *M. camini* DV1197, respectively. The versions described in this paper are the first versions, AZAX01000000 and AZAY01000000.
